# Environmental and maternal factors shaping tonsillar microbiota development in piglets

**DOI:** 10.1186/s12866-022-02625-8

**Published:** 2022-09-26

**Authors:** Simen Fredriksen, Xiaonan Guan, Jos Boekhorst, Francesc Molist, Peter van Baarlen, Jerry M. Wells

**Affiliations:** 1 Host-Microbe Interactomics Group, Animal Sciences Department, Wageningen University, Wageningen, The Netherlands; 2 Schothorst Feed Research B.V, Lelystad, The Netherlands; 3 Department of Veterinary Medicine, University of Cambridge, Cambridge, U.K.

**Keywords:** piglet, microbiota, early life, longitudinal, sow

## Abstract

**Background:**

The palatine tonsils are part of the mucosal immune system and stimulate immune responses through M cell uptake sampling of antigens and bacteria in the tonsillar crypts. Little is known about the development of the tonsillar microbiota and the factors determining the establishment and proliferation of disease-associated bacteria such as *Streptococcus suis*. In this study, we assessed tonsillar microbiota development in piglets during the first 5 weeks of life and identified the relative importance of maternal and environmental farm parameters influencing the tonsillar microbiota at different ages. Additionally, we studied the effect sow vaccination with a bacterin against *S. suis* on microbiota development and *S. suis* colonisation in their offspring.

**Results:**

Amplicon sequencing of the 16S rRNA gene V3-V4 region revealed that a diverse tonsillar microbiota is established shortly after birth, which then gradually changes during the first 5 weeks of life without a large impact of weaning on composition or diversity. We found a strong litter effect, with siblings sharing a more similar microbiota compared to non-sibling piglets. Co-housing in rooms, within which litters were housed in separate pens, also had a large impact on microbiota composition. Sow parity and prepartum *S. suis* bacterin vaccination of sows had weaker but significant associations with microbiota composition, impacting on the abundance of *Streptococcus* species before and after weaning. Sex and birthweight had limited impact on the tonsillar microbiota, and none of the measured factors had consistent associations with microbiota diversity.

**Conclusions:**

The piglet tonsillar microbiota is established shortly after birth. While microbiota development is associated with both environmental and maternal parameters, weaning has limited impact on microbiota composition. Intramuscular vaccination of sows pre-partum had a significant effect on the tonsillar microbiota composition of their piglets. These findings provide new insights into the mechanisms shaping the tonsillar microbiota.

**Supplementary Information:**

The online version contains supplementary material available at 10.1186/s12866-022-02625-8.

## Introduction

The palatine tonsils contain lymphoid tissue covered by a stratified squamous epithelium that extends into tonsillar crypts. M cells located in the crypt epithelial layer sample environmental antigens, including bacteria, for presentation by resident dendritic cells or macrophages and induction of immune (IgA) responses in saliva and the respiratory tract [1–3]. The tonsillar microbiota is therefore a major stimulus for mucosal immune responses but can also harbour pathogens which may use the tonsils as a portal of entry into the host [4].

A better understanding of the tonsillar microbiota and factors influencing its development is of particular importance in pigs due to colonization by and persistence of pathogenic bacteria such as *Actinobacillus pleuropneumoniae* [5], *Salmonella enterica* [6], and *Streptococcus suis* [7]. *Streptococcus suis* is especially relevant to microbiota research because of its high carriage rates in healthy piglets and ability to cause sepsis, meningitis, and arthritis [8–10]. Previous studies have found a large increase in the relative abundance of the family *Streptococcaceae* after weaning, coinciding with the abrupt change in piglet diet from milk to starch-rich dry feed at weaning [11–13]. These studies used antimicrobial growth promoters, and the described microbiota development may vary from Europe where these are banned. Regardless, the increase in *Streptococcaceae* family abundance is of interest because correlations between *S. suis*, carbohydrate availability, and microbiota composition have been linked to *S. suis* virulence and disease risk [9, 10, 14–16].

There are no cross-protective vaccines against *S. suis*, but vaccination of sows with autogenous bacterins, vaccines made of dead cells of cultured clinical strains isolated from the individual farm, is used to provide passive immunity against *S. suis* in piglets. Their effectiveness is a matter of debate [9, 17–20]. A recent study showed no lasting protection of piglets after sow vaccination, and vaccination of piglets failed to induce an active immune response [21]. The effect of bacterin vaccination on *S. suis* colonisation and tonsil microbiota development is less well described. In humans, a meta-analysis on infants vaccinated against *S. pneumoniae* showed a reduced carriage of the vaccine capsule types [22], but intramuscular bacterin vaccination of piglets has shown no effect on *S. suis* colonisation [21, 23]. Systemic vaccines rarely impact on mucosal immunity and colonization [24], but vaccination of sows may also impact on *S. suis* colonisation and the tonsil microbiota in their offspring via opsonising antibodies in colostrum and milk.

The aim of this study was to investigate the impact of maternal and environmental effects on the neonate tonsillar microbiota development. We utilized 16S rRNA gene V3-V4 region amplicon sequencing to assess the microbiota composition of 63 piglets from 21 different litters shortly after birth and at week 1, 3 and 5. One group of sows was injected with a multi-strain *S. suis* autogenous bacterin prior to farrowing, allowing us to assess the effects of sow vaccination on the microbiota of their offspring.

## Methods

### Experimental design

This study was carried out at Schothorst Feed Research BV, a high-health status research farm in the Netherlands. We selected 21 sows of varying parity (including 1 gilt) with normal body condition for the study (Table S1). Eighteen of the sows were Landrace x Large White, the remaining 3 sows Large White x Large White. All piglets were from the same sire line: Tempo (TOPIGS Norsvin). For analysis, sows were grouped as young parity (parity 0, 1, and 2) or old parity (parity 4, 5, and 6). Nine sows received two 2 mL (between 10^7^ and 10^8^ cfu/mL) intramuscular injections of a bacterin vaccine approximately six and two weeks before farrowing, while 12 unvaccinated sows served as controls. No piglets received the vaccine. The bacterin was prepared from two *Streptococcus suis* strains (serotypes 7 and 9) isolated from autopsy cases of *S. suis* invasive disease on the same farm. The bacterin was made by Dopharma BV (The Netherlands).

All sows were inseminated on the same day. Sows were housed separately, one sow per pen, in three different farrowing rooms from day 109 of gestation. Each room housed 8 pens, each with a size of 0.60 × 2.50 m for the sow and 2.25 × 2.50 m total surface. Three litters from one of the rooms (room 5) were not included for microbiota sequencing. These litters were, however, also a part of the experimental design and no known factors may have influenced the microbiota in this room differently to the other rooms. Large litters were reduced in size by cross-fostering within 48 h after birth, but no litters in the study received piglets from another sow. Of the total 362 piglets born, 28 were born dead, 15 cross-fostered, and 49 died before weaning. Creep feed was provided from 7 days after birth until weaning. See Table S2 for the content of all feed types used in the study. Piglets were weaned at approximately 26 days of age, and litters were moved to nursery pens (2.00 × 1.13 m) each containing 6 piglets, with the piglets selected for microbiota sampling remaining co-housed to avoid microbiota transfer. This study design did not allow determination of the post-weaning room and pen effect.

Three piglets from each litter were selected for microbiota sequencing based on birth weight and avoiding piglets that died or developed disease symptoms throughout the study period (Table S3). We selected one low birth weight (LBW), one median birth weight (NBW), and one high birth weight (HBW) piglet per litter. Birth weight varied between litters, meaning that the weight of some LBW piglets was heavier than NBW or HBW piglets of other litters. Piglets were first sampled with tonsil swabs and weighed as soon as possible after birth. Because piglets were born over several days, and only handled during working hours, this occurred separately per litter and up to 48 h after birth. The study cohort was next sampled all at the same time at week 1 after birth, the timepoint being set as the average age of all piglets. This meant that some piglets were 4 days old and others 9 days old. Subsequently, all piglets were sampled at week 3. At week 4, all piglets were weaned and weighed. One week later, at week 5, all piglets were sampled for the last timepoint for microbiota sampling. At week 7, all piglets were again weighed, but not sampled, before being mixed and utilized in other studies. Because of confounding treatments in these studies, we calculated post-weaning growth rate only for the first 3 weeks post-weaning.

To compare the piglet microbiota with that from adult sows we collected tonsil swab samples from 12 unrelated sows from the same farm at slaughter. We did not collect tonsil swabs from the original sows used in the study because these sows were not sacrificed, and sampling from living sows requires anaesthesia treatment that would have interfered with the experiment.

### Microbiota sequencing

We sampled the tonsillar microbiota of piglets by gently scraping the palatine tonsils with a HydraFlock swab (Puritan, USA) for 10 s. Swabs were placed in vials containing Powerbead solution (Qiagen, The Netherlands) and stored at -80 °C. DNA was isolated using the PowerSoil DNA Isolation Kit (Qiagen) with 0.7 mm garnet beads. DNA yield was similar between timepoints (week 0: 44 ng/µl, SD = 27; week 1: 40 ng/µl, SD = 23; week 3: 48 ng/µl, SD = 24; week 5: 34 ng/µl, SD = 22). Subsequently the V3-V4 region of the 16S rRNA gene was amplified with primers 341F (5′-CCTAYGGGRBGCASCAG-3′) and 806R (5′-GGACTACNNGGGTATCTAAT-3′), using 35 PCR cycles with Phusion High-Fidelity PCR Master Mix (New England Biolabs, USA). DNA libraries were prepared with the NEBNext Ultra II DNA Library Prep Kit. DNA quantity and fragment size was checked with Qubit and BioAnalyzer before 250 bp paired-end sequencing on an Illumina NovaSeq 6000 machine. ZYMO mock community #D6300/#D6305 was used to verify the accuracy of our protocol and we processed blank swabs together with the tonsil microbiota samples as controls. Analysis of the abundance of the amplicon sequence variants (ASVs) identical to the mock community members 16S rRNA gene V3-V4 region showed that DNA from all community members was successfully isolated and amplified. We found a moderate bias towards disproportionally high abundance of gram negatives *Escherichia coli* and *Salmonella enterica*, while other species were underrepresented (Figure S1). Three blank control swabs processed together with the tonsil microbiota samples produced no visible bands following PCR amplification.

After sequencing, primers were trimmed with Cutadapt 2.3 [25] and processed in DADA2 [26] with taxonomic assignment to SILVA database v138 [27]. Amplicon sequence variants with taxonomic assignment as eukaryote, chloroplast, or mitochondria were discarded before carrying out further analyses. The ASV abundance per sample was rarefied to the sample with the lowest read count (42,537 reads). R packages Phyloseq [28] and vegan [29] were used to calculate alpha and beta diversity. All results shown in the main text are based on Shannon diversity and Bray–Curtis dissimilarity. Vegan function RDA was used for principal component analysis (PCA) and redundancy analysis (RDA), and function adonis was used for permutational analysis of variance (PERMANOVA). We used LEfSe (Linear discriminant analysis Effect Size) [30] to find taxa associated with different timepoints. ASV correlation with numerical variables (such as growth rates and parity) was calculated with Spearman’s rank correlation coefficient, while Wilcoxon Rank Sum Test was used for categorical variables (such as sow vaccination and pairwise comparison of timepoints).

A previous longitudinal study on the piglet tonsillar microbiota [13] reported taxonomy only at the family level. We reanalysed their dataset (BioProject PRJNA391812) with the approach used in the present study as described above. The reanalysed study used different primers, sequencing the 16S rRNA gene V4 region while we used the V3-V4 region. Both primer sets did, however, allow us to distinguish between the two species of interest, *S. suis* and *S. porcorum*, in SILVA database v138.

### In vitro growth experiment

Carbon source utilization of *Streptococcus* species from the oral cavity of pigs was tested in vitro by measuring growth in a complex medium (CM) with defined carbon sources. The complex medium was made as previously described [31] and supplemented with 0.2 μm filter sterilized D-glucose, lactose, maltotriose, or pullulan at 0.2 g/100 mL as carbon source. Overnight cultures were prepared in Todd-Hewitt yeast broth (THY) inoculated with single colonies from THY agar plates and diluted 1:50 in PBS. 10 μL diluted overnight culture was inoculated in 190 μL fresh THY medium in 96-well plates. In total, six plates each containing 2 replicates of each treatment were incubated at 37 °C for 15 h and optical density measured at 600 nm every 15 min using a SpectraMax M5 (Molecular Devices) spectrophotometer.

## Results

### Overview of the tonsillar microbiota composition

We sequenced the tonsillar microbiota of 63 piglets from 21 different litters with 16S rRNA gene V3-V4 region amplicon sequencing. Tonsil microbiota samples were collected within 48 h after birth, and when the average age of the cohort was of 1, 3, and 5 weeks old. Nine of the 21 sows were vaccinated with a *S. suis* bacterin vaccine 6 and 2 weeks before expected parturition. All samples were successfully processed and 250 bp paired-end sequenced, resulting in 252 piglet and 12 sow tonsillar microbiota samples with a minimum of 42,537 reads and in total 25,267 ASVs after processing with DADA2 (Figure S2).

**Fig. 1:**
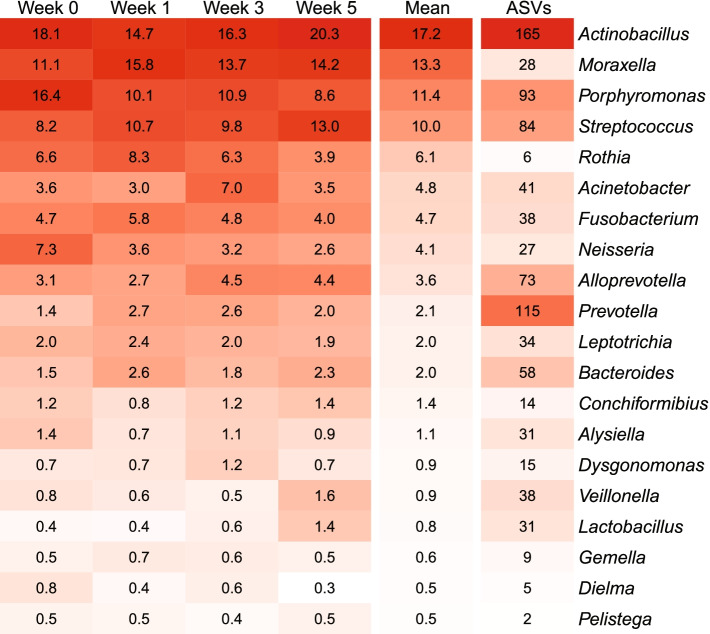
Heatmap showing relative abundance (%) of the most abundant genera at different timepoints and the number of ASVs with more than 100 reads for each genus

The most abundant genera of the piglet tonsillar microbiota were *Actinobacillus*/*Haemophilus* (two abundant genera indistinguishable with 16S rRNA gene V3-V4 region amplicons), *Moraxella*, *Porphyromonas*, and *Streptococcus*; these genera exceeded 10% mean abundance across the dataset. *Rothia*, *Fusobacterium*, *Neisseria*, *Alloprevotella*, and *Acinetobacter* each contributed more than 3% abundance (Fig. 1). Figure S3 shows the abundance of the most abundant genera per sample. Most of the abundant genera were represented by a large number of different ASVs (Fig. 1), indicating that the tonsillar microbiota is colonized by multiple closely related strains. Nineteen ASVs contributed 1% abundance or more, and 167 ASVs were present at 0.1% or higher abundance (Table S4). Thirty four of these 167 ASVs were classified as *Actinobacillus*/*Haemophilus*, 21 as *Porphyromonas*, and 15 as *Streptococcus*. Three of the six most abundant ASVs were classified as *Moraxella* and differed by only a single SNP.

### Tonsillar microbiota development during the first 5 weeks of life

**Fig. 2:**
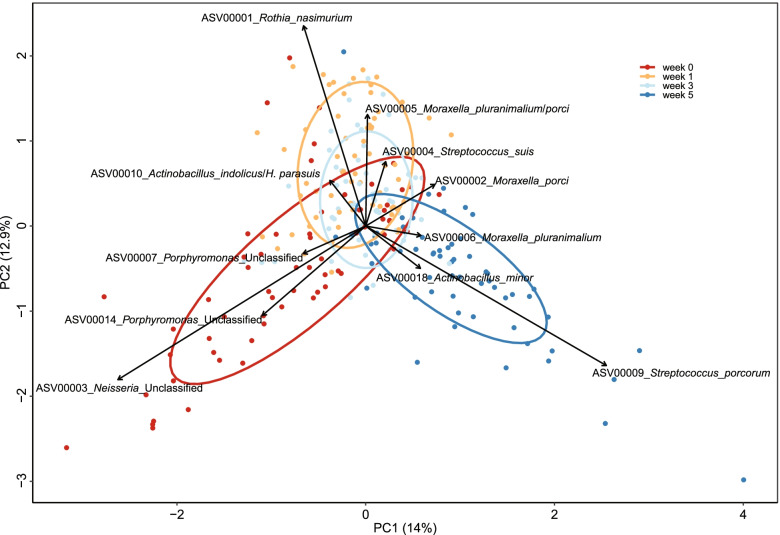
PCA plot showing differences in tonsillar microbiota composition by age. Arrows show ASVs driving the separation; samples in the direction the arrow is pointing have higher abundance of that ASV. Each point represents a sample. At the first timepoint (week 0), the tonsillar microbiota was characterised by a high abundance of *Neisseria* and *Porphyromonas* ASVs, while post-weaning samples (week 5) had a high abundance of *Streptococcus porcorum*

Microbiota composition changed with age (PERMANOVA; R2 = 0.13, *p* < 0.001), but the age effect was not strong enough to fully separate samples by timepoint with PCA (Fig. 2). At the initial timepoint (week 0) we found high abundance of *Porphyromonas* sp. and *Neisseri*a sp. ASVs, but these ASVs decreased in abundance before week 1 (*Neisseri*a ASV 3 from 7.3 to 3.4%, *Porphyromonas* ASV 7 and ASV 14 from 3.9 to 2.1% and 2.6 to 0.66%, respectively). These 3 ASVs all had weak negative correlations with birthweight at the first timepoints. *Neisseri*a ASV 3 had the strongest negative correlation with birthweight at the initial sampling (*R* = -0.19, *p* = 0.13), while *Porphyromonas* ASVs 7 and 14 had stronger correlations at week 1 (*R* = -0.31, *p* = 0.01 and -0.15, *p* = -0.24, respectively).

At week 1 the abundance of ASV 1 (*Rothia nasimurium*), *Moraxella pluranimalium*/*porci* (ASV *2* and ASV 5), and *Streptococcus suis* (ASV 4) increased compared to week 0 (4.1 to 6.4%, 3.4 to 4.6%, 2.5 to 5.7%, and 3.1 to 5.0%, respectively). LEfSe analysis on the abundance of taxa at different timepoints also found that *Porphyromonas* and *Neisseria* were associated with the initial timepoint, *Rothia* and *Moraxella* with week 1, and *Streptococcus* species with week 5 (Figure S4).

Piglets were weaned at week 4, at approximately 26 days old, changing their diet from sow milk with creep feed supplementation to starch-rich dry feed. This might be expected to reshape the microbiota, in part due to the change in available carbohydrates. Between week 3 and 5 the most abundant *Streptococcus suis* ASV (ASV 4) decreased in abundance (3.9 to 2.4%), while ASV 9 (*Streptococcus porcorum)* increased from 0.8 to 5.5% abundance, becoming the most abundant ASV at week 5. Several other less abundant *Streptococcus* ASVs also increased in abundance between week 3 and 5, including ASV 445 (*Streptococcus caballi*, 0.007 to 0.03%), ASV 97 (*Streptococcus hyointestinalis*, 0.1 to 0.6%), and ASV 195 (*Streptococcus porci*, 0.04 to 0.21%).

Overall change in microbiota composition measured by Bray–Curtis dissimilarity was, however, similar between timepoints. The smallest change occurred between week 1 and 3 (mean Bray–Curtis dissimilarity = 0.46), the second smallest change occurred between week 0 and 1 (mean = 0.48), and the largest between week 3 and 5 (mean = 0.49). The number of the most abundant ASVs changing in abundance was also similar between timepoints. Of the 167 ASVs with 0.1% or higher overall abundance, 72, 66, and 72 significantly changed in abundance between timepoints 0 to 1, 1 to 3, and 3 to 5 respectively. The impact of weaning was more evident on rare ASVs. Among the 3724 ASVs with 0.001% or higher overall abundance, 69, 103, and 134 ASVs changed in abundance between timepoints 0 to 1, 1 to 3, and 3 to 5 (Wilcoxon Rank Sum test FDR < 0.05).

A previous study on the piglet tonsillar microbiota in the USA [13] found a similar but stronger increase in overall *Streptococcacae* abundance after weaning but did not report changes at the species level. To evaluate whether *S. porcorum* was responsible for the increase in *Streptococcacae* we re-analysed their dataset (BioProject PRJNA391812) with the approach used in the present study. Our reanalysis found a strong *S. porcorum* increase across weaning, from 0.1 to 29.8%. The increase in *S. porcorum* coincided with an overall decrease in *S. suis* from 5.0 to 2.8%, similar to the decrease from 6.1 to 4.6% in the present study (Figure S5).

**Fig. 3:**
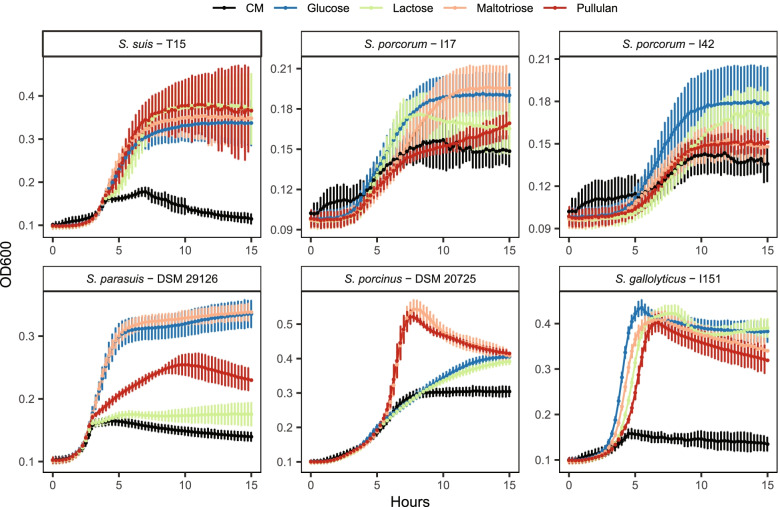
Growth curves (note: independent y-axis per strain) of 6 strains from five *Streptococcus* species found in the oral cavity of pigs on complex media (CM) supplemented with different carbohydrate sources. While growth curves differ between strains, all screened strains reached higher OD_600_ values with all added carbohydrates than CM alone

To determine whether the decrease in *S. suis* and increase of *S. porcorum* might be related to carbohydrate utilisation we conducted an in vitro growth experiment in complex medium (CM) containing different carbohydrates present before and/or after weaning. We included 6 different strains of 5 *Streptococcus* species found in the oral cavity of pigs. All strains grew to higher OD_600_ values with all added carbohydrates compared to base complex media (CM) alone, showing that all the tested strains can utilize lactose (present in sow milk), maltotriose (produced by the breakdown of starch by salivary amylase), and the starch dextran pullulan (Fig. 3). Some strains did, however, reach higher OD_600_ values on specific carbon sources. *S. parasuis* reached higher OD_600_ values on glucose and maltotriose compared to pullulan and lactose, and *S. porcinus* grew more rapidly to high OD_600_ values on maltotriose and pullulan compared to the other media. The two *S. porcorum* strains differed in the growth on lactose and pullulan. Although we cannot ascertain the phenotype of the strains present *in-vivo*, the tested *S. porcorum* and *S. suis* strains grew on lactose, maltotriose, and pullulan in vitro*,* suggesting that factors other than availability of lactose and starch explain the change in abundance after weaning.

### Alpha diversity increases during the first 5 weeks of life

**Fig. 4:**
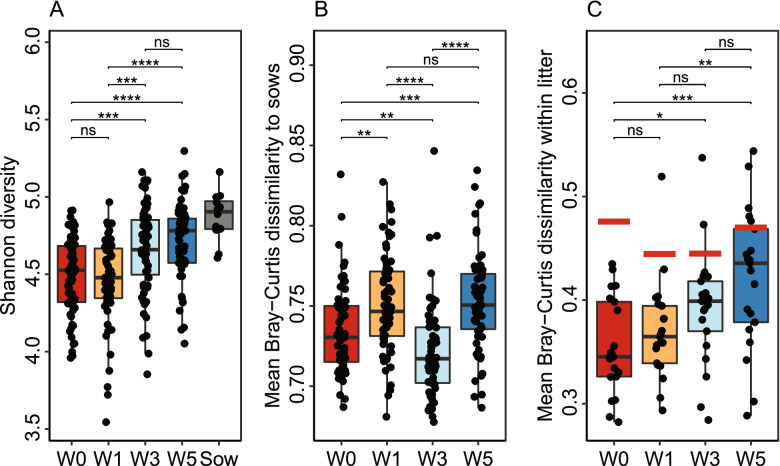
**a **Shannon diversity increases with age and approaches adult sow microbiota diversity. **b** Mean Bray–Curtis dissimilarity between piglets and unrelated sows on the same farm. Significance calculated by Wilcoxon Rank Sum Test is indicated, * *p* < 0.05, ** *p* < 0.01, *** *p* < 0.001, **** *p *< 0.0001. **c**) Total litter effect measured in mean Bray–Curtis dissimilarity between siblings at each timepoint. While each point in **a**) and **b**) represents a single piglet, each point in **c**) represents a litter, and its y-axis value is the mean of all possible pairwise comparisons between the siblings. Red horizontal lines indicate the mean dissimilarity between all piglets at the timepoint. The mean dissimilarity between all piglets was comparable to that between the unrelated sows (mean = 0.48, not shown)

Alpha diversity increased with age but did not reach as high Shannon diversity as sows by week 5 (Fig. 4a). The diversity increase with age was consistent between alpha diversity metrics, but although sows had high alpha diversity their ASV richness was lower (Figure S6). To assess the impact of different factors on microbiota diversity, we used a multivariate ANOVA on Shannon diversity with the model BW_kg (numeric BW) + BW_relative (low, median, or high BW within litter) + Sex + Farrowing_room + Parity_group (0, 1 and 2 vs 4, 5, and 6) + Sow_vaccination + Sow_ID + Daily_growth_pre_weaning + Daily_growth_post_weaning for each timepoint. None of the included factors were consistently associated with alpha diversity over time (Table S5). Birth weight had a small impact on diversity directly after birth (relative within litter: *p* < 0.05, absolute: *p* = 0.09 at week 0), but this association decreased with age. Pre-partum sow vaccination also had the strongest association with alpha diversity at the first timepoint (*p* = 0.18). At the later timepoints, the farrowing room, sow parity, and the individual sow were significant factors (*p* < 0.05) at one or more timepoints. These factors all saw a dip in significance at week 3 before increasing to week 5. Pre- and post-weaning growth rate was not significantly (*p* < 0.05) associated with diversity at any timepoint, although diversity at week 1 had a positive correlation with post-weaning growth rate (*p* = 0.051). Redundancy analysis (RDA) on microbiota composition with growth rate as constraint did not reveal any significant interaction, and no individual ASV had a significant Spearman correlation with growth rate at any timepoint (FDR < 0.05).

### Comparison of the tonsillar microbiota composition in piglets and sows

The compositional changes over the 5 first weeks of piglet life did not show any signs of converging towards the adult sow tonsillar microbiota (Fig. 4b) and remained significantly different at week 5 (PERMANOVA; R2 = 0.30, *p* < 0.001). The sow tonsillar microbiota differed from that of piglets most notably by lower abundance of *Rothia* (4.1% vs 1.5%), *Streptococcus* (13.6% vs 2.3%), and *Moraxella* (13.8% vs 7.2%) and higher abundance of *Acinetobacter* (3.7% vs 16.7%), *Conchiformibius* (1.5% vs 8.5%), and *Alysiella* (0.87% vs 4.1%) (all FDR < 0.01; Table S4). Most abundant ASVs in the piglet tonsillar microbiota were present in the sow tonsillar microbiota, although at lower abundance. The most abundant ASVs not present in the sow tonsillar samples were ASV 5 (*Moraxella pluranimalium*/*porci*), ASV 19 (*Moraxella* sp.), ASV 23 (*Actinobacillus porcinus*), and ASV 22 (*Rothia nasimurium*). *Streptococcus porcorum* abundance was also low in sows (mean: 0.02%).

### Statistical associations between the tonsillar microbiota and farm parameters

**Fig. 5:**
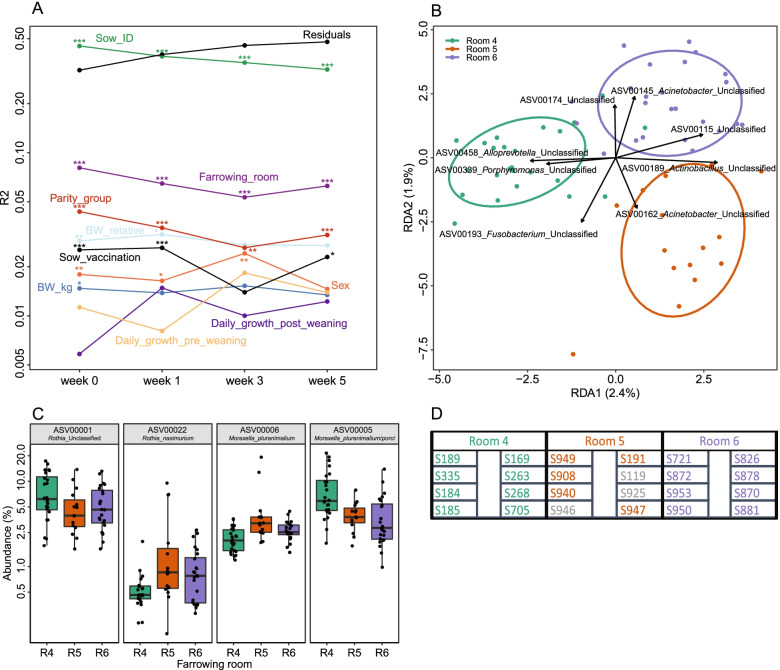
**a** The additive effect of factors on the piglet tonsillar microbiota composition calculated by PERMANOVA on Bray–Curtis dissimilarity. Most variation in the dataset remains unexplained by the included factors, as shown in the high values of Sow_ID and residuals (i.e., variation specific to the individual sow (litter) or piglet and not explained by the other factors). Statistical significance is indicated, * *p* < 0.05, ** *p* < 0.01, *** *p* < 0.001. **b** The farrowing room effect at week 1 shown by RDA, abundance transformed by log(1000*abundance + 1). **c** The abundance of the 4 strongest drivers of farrowing room separation in RDA without abundance transformation. **d** Schematic presentation of the rooms and pens. 3 litters from room 5 were not included for microbiota sequencing (marked in grey)

The tonsillar microbiota composition of piglets may be influenced by a range of factors, and we observed a strong litter effect, with a lower mean Bray–Curtis dissimilarity between siblings than between random piglets (Fig. 4c). This overall litter effect may involve a range of factors linked to both the sow and shared environment. We determined the associations between microbiota composition and all measured parameters with PERMANOVA on Bray–Curtis dissimilarity at each timepoint with the model described above for alpha diversity (Fig. 5a). For PERMANOVA results with different beta-diversity metrics see Table S6. The farrowing room played a significant role (*p* < 0.01), starting at R2 = 0.08 after birth and remaining at R2 = 0.06 at week 5 (Fig. 5b-d). Sow parity and pre-partum vaccination had smaller but significant associations (*p* < 0.01 at week 1). As for alpha diversity, the effect of parity, sow vaccination, and farrowing room decreased in strength at week 3 before recovering at week 5. Birth weight had a small but significant (*p* < 0.05) association both measured in absolute terms and relative to littermates, but this association decreased with age. The sow ID, in the model representing residual litter effect not explained by the previous factors, was the strongest factor at week 0 (R2 = 0.45, *p* < 0.001). The variation explained by the individual sow decreased between each timepoint but remained the strongest factor at week 5 (R2 = 0.32, *p* < 0.01).

To further investigate associations between microbiota composition and the model variables we used RDA on each variable for each timepoint. This analysis confirmed the findings from the PERMANOVA, with strong associations between microbiota composition and farrowing room and sow parity (maximum RDA1 = 5.7 and 4.9%, respectively). Absolute and relative birth weight and sex had weaker and non-significant associations (RDA1 < 2% and *p* > 0.2). Sow vaccination remained significant at week 1 and 3 (RDA1 = 3.3 and 3.2%, respectively).

While the overall microbiota had significant associations with several factors with RDA and measured in Bray–Curtis dissimilarity, few specific ASVs had consistent associations between timepoints. The microbiota difference between farrowing rooms were not caused by ASV presence/absence, but different abundance of shared ASVs (Fig. 5B-C).

### Bacterin vaccination altered the abundance of *Streptococcus* species

**Fig. 6:**
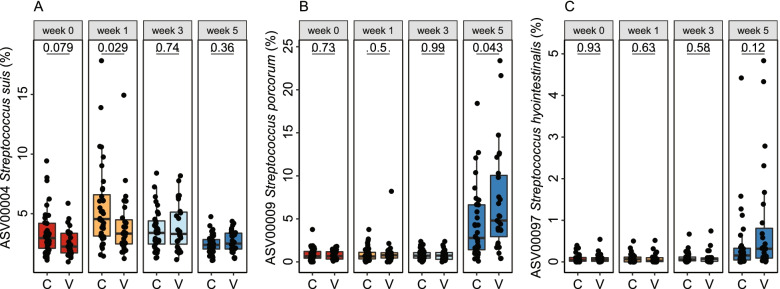
Sow vaccination altered the abundance of *Streptococcus* species. ASV 4 (*S. suis* ASV matching the 16S rRNA gene of the bacterin vaccine strains) abundance was lower in piglets of vaccinated sows (V) than controls (**C**) at the first two timepoints (**A**), while (**B**) ASV 9 (*S. porcorum*) and (**C**) ASV 97 (*S. hyointestinalis*) were more abundant in the control group post-weaning (**B**). Wilcoxon Rank Sum Test p-values are shown

Pre-partum vaccination of sows with a *S. suis* bacterin affected the microbiota composition of their piglets (PERMANOVA; R2 = 0.026, *p* < 0.05 at week 1). This was in part driven by differences in the abundance of ASV 4 (*S. suis*); this ASV matched the 16S rRNA gene of both bacterin strains at 100% identity. ASV 4 was less abundant in the piglets of vaccinated sows during the first week of life, but by week 3 its abundance was similar to the control group (Fig. 6). The overall difference in microbiota composition was again significant at week 5, after weaning (PERMANOVA; R2 = 0.026, *p* < 0.05). This was driven in part by a higher abundance of ASV 9 (*S. porcorum,* 4.31% vs 6.96%) and ASV 97 (*S. hyointestinalis*, 0.39% vs 0.83%). The vaccine effect was additive with a parity effect on the same ASVs; the vaccine effect occurred within both high- and low-parity sows. For instance, ASV 4 (*S. suis*) abundance at week 1 was 3.64% in vaccinated high-parity litters, 4.79% for [no vaccine + high parity], 4.47% for [vaccine + low parity], and 7.10% for [no vaccine + low parity].

## Discussion

In this study, we followed changes in the tonsillar microbiota of piglets from shortly after birth until one week after weaning. We investigated the association between tonsillar microbiota composition and diversity and environmental, maternal, and individual piglet parameters. We found a strong litter effect, i.e., piglets born by the same sow sharing a similar microbiota. This is in agreement with previous studies on the oral [13, 32] and gut [33, 34] microbiota of piglets.

The litter effect on the piglet microbiota may be caused by a variety of factors. For example, environmental factors related to the pen and microbiota transfer from the environment and via aerosols [35] may be important determinants of microbiota composition. Horizontal microbiota transfer between siblings likely also plays a large role. In this study, strong environmental effects were indicated by the large effect of the farrowing rooms, where several litters were housed in separate pens. The farrowing room differences were not caused by presence/absence of different taxa, but small differences in abundance of shared ASV. These differences may be stochastic and driven by microbiota transmission within rooms. Pens were divided with solid separators prevented direct contact between piglets in neighbouring pens, but airborne transfer is possible [35, 36]. A previous study found inoculated *S. suis* serotype 9 to transmit between separated pens, albeit significantly slower than via direct contact [36]. While the environment on the research farm used for this study is controlled, small differences in temperature or ventilation between the rooms may also have played a role. Further research on this topic is warranted.

Sow factors such as genetics, behaviour, milk and colostrum content, and vertical microbiota transfer from sow to litter may also cause litter effects, as shown by the impact of sow parity and pre-partum vaccination of sows. This is consistent with a previous study showing that the nasal microbiota of piglets is strongly influenced by sow contact [32]. Parity has been reported to influence nasal and faecal microbiota composition of piglets [15, 33], and is positively associated with piglet performance and negatively with piglet mortality [37].

We found a weak association between tonsillar microbiota composition and birthweight. Previous work on the gut microbiota of piglets have reported stronger associations [38–41], and linked this to the effects of intrauterine growth restriction. Intrauterine growth restriction and/or low birth weight has been associated with improper development of intestinal mucosal immunity [42] and gut epithelial barrier function [43], as well as reduced colostrum, milk, and creep feed intake [37, 44–46]. The weaker association between birthweight and the tonsillar microbiota may be due to less developmental impact of intrauterine growth restriction on the tonsils compared to the gut, but also due to the tonsillar microbiota being less influenced by milk and feed intake compared to gut microbiota.

Prepartum vaccination of sows with a *S. suis* bacterin impacted the tonsillar microbiota composition of their piglets, initially by reducing the abundance of the *S. suis* ASV matching the strains used in the vaccine during the first week after birth. A recent study found similar effects of pre-partum sow vaccination on the piglet nasal microbiota and *Glaesserella parasuis* abundance [47]. The vaccination effect may be due to alterations in the vertically transferred sow microbiota, although intramuscular vaccination rarely impacts mucosal immunity [24]. It is also possible that maternal antibodies in colostrum and milk from vaccinated sows influence *S. suis* persistence through immune exclusion. Apart from the effect of the sow vaccination on *S. suis*, the tonsil microbiota composition of piglets was significantly different to the control group also after weaning, at week 5, in part due to higher abundance of *S. porcorum* and *S. hyointestinalis*. More complex and indirect microbe-microbe interactions may underlie the higher *Streptococcus* abundance post-weaning, as the piglets had stopped suckling. Future studies may want to include analysis of sow microbiota and antibody titres to better assess the mechanisms behind the strong sow influence on the piglet microbiota.

*Streptococcus porcorum* also stood out as the main species increasing in abundance after weaning, while *S. suis* abundance decreased. *Streptococcus porcorum* is closely related to *S. suis* but has shown differential efficiency in carbohydrate utilisation [48]. We considered it possible that *S. porcorum* might have gained a competitive advantage upon the increased availability of starch after weaning, but an in vitro experiment showed that neither *S. porcorum* nor *S. suis* gained a distinct advantage when provided lactose, maltotriose (available in the oral cavity after breakdown of starch by host amylase activity), or the starch dextran pullulan as carbon sources. Thus, the mechanism behind the large increase in *S. porcorum* abundance remains unknown.

Contrary to previous work on both the tonsillar [13] and gut microbiota [49–51], there was no striking change in the microbiota composition after weaning, indicating that the impact of host diet on tonsillar microbiota composition is modest. The larger change in tonsillar microbiota composition previously found [13] was also largely driven by *S. porcorum*, but the increase and change in the overall microbiota composition was far larger. This might be explained by piglets in the present study having access to pre-weaning creep feed, allowing post-weaning-associated bacteria to gain an early foothold, and by the previous study including feed supplementation with the antimicrobial Carbadox. The persistence of a relatively undisturbed microbiota in this study may also have contributed to persistence of the strong litter effect after weaning.

## Conclusions

Our results show that the tonsillar microbiota of piglets is established within the first days after birth, and that microbiota composition and diversity changes gradually during the first five weeks of life without converging on the adult sow tonsillar microbiota composition. The impact of weaning and host diet change on the tonsillar microbiota appears to be limited. Tonsillar microbiota composition was associated with a dominant litter effect linked to both environmental and maternal factors. Prepartum *S. suis* bacterin vaccination of sows resulted in reduced abundance of *S. suis* in the tonsillar microbiota in litters of vaccinated sows. We considered that this effect was most likely due to immune exclusion from anti-*S. suis* antibodies in colostrum and sow milk saturating the tonsil surface. Piglets of vaccinated sows also showed significantly altered microbiota composition and increased *S. porcorum* abundance post-weaning, suggesting that the bacterin vaccination had fundamentally altered microbiota development. Our findings show promise for the potential of microbiome manipulation by probiotics and vaccination strategies to avoid infectious disease in young piglets.

## Supplementary Information

**Additional file 1: Table S1.** Metadata on the sows included in the study.

**Additional file 2: Table S2.** Detailed content of the ingredients used in the different diets used in the study.

**Additional file 3: Table S3.** Metadata on the piglets included in the study.

**Additional file 4: Table S4**. Overview of the 1000 most abundant ASVs, their sequence, taxonomy, and abundance at the different timepoints and in adult sows.

**Additional file 5: Table S5.** ANOVA results for Shannon, InvSimpson, Chao1, and PD alpha diversity metrics.

**Additional file 6: Table S6.** PERMANOVA results for Bray-Curtis, weighted and unweighted UniFrac, and Jaccard beta diversity metrics.

**Additional file 7:** **Figure S1.** Boxplot showing the relative abundance of ASVs with 100% identity to the 16S rRNA gene V3-V4 region of mock community members. We used both a DNA isolation mock community (ZymoBIOMICS Microbial Community Standard ZYMO #D6300) and PCR mock community ZymoBIOMICS Microbial Community DNA Standard ZYMO #D6305).

**Additional file 8: Figure S2.** Rarefaction curves for all samples. Constructed with Vegan function rarecurve.

**Additional file 9: Figure S3.** Stacked barplot showing the abundance of the most abundant genera in all samples.

**Additional file 10: Figure S4.** LEfSe analysis on taxa association with timepoints.

**Additional file 11: Figure S5.** Comparison of *S. suis* and *S. porcorum* abundance at different timepoints in the present study and Pena Cortes et. al. 2018 (NCBI BioProject PRJNA391812). *S. suis* abundance decreases before and across weaning, while *S. porcorum* increases in abundance at weaning. The higher number of zero-counts found in the dataset of Cortes et al. is in part due to larger variation in sequencing depth. 12 samples had less than 1000 reads.

**Additional file 12: Figure S6.** Boxplots of different alpha diversity measures per timepoint.

## Data Availability

All sequencing data generated in the current study are available in the DANS repository at https://doi.org/10.17026/dans-xxb-56zp.
