# Supplementary figures and images for: Environmental and maternal factors shaping tonsillar microbiota development in piglets
Source: BMC Microbiol. 2022 Sep 26;22:224. doi: 10.1186/s12866-022-02625-8 (PMC9513891; doi:10.1186/s12866-022-02625-8)

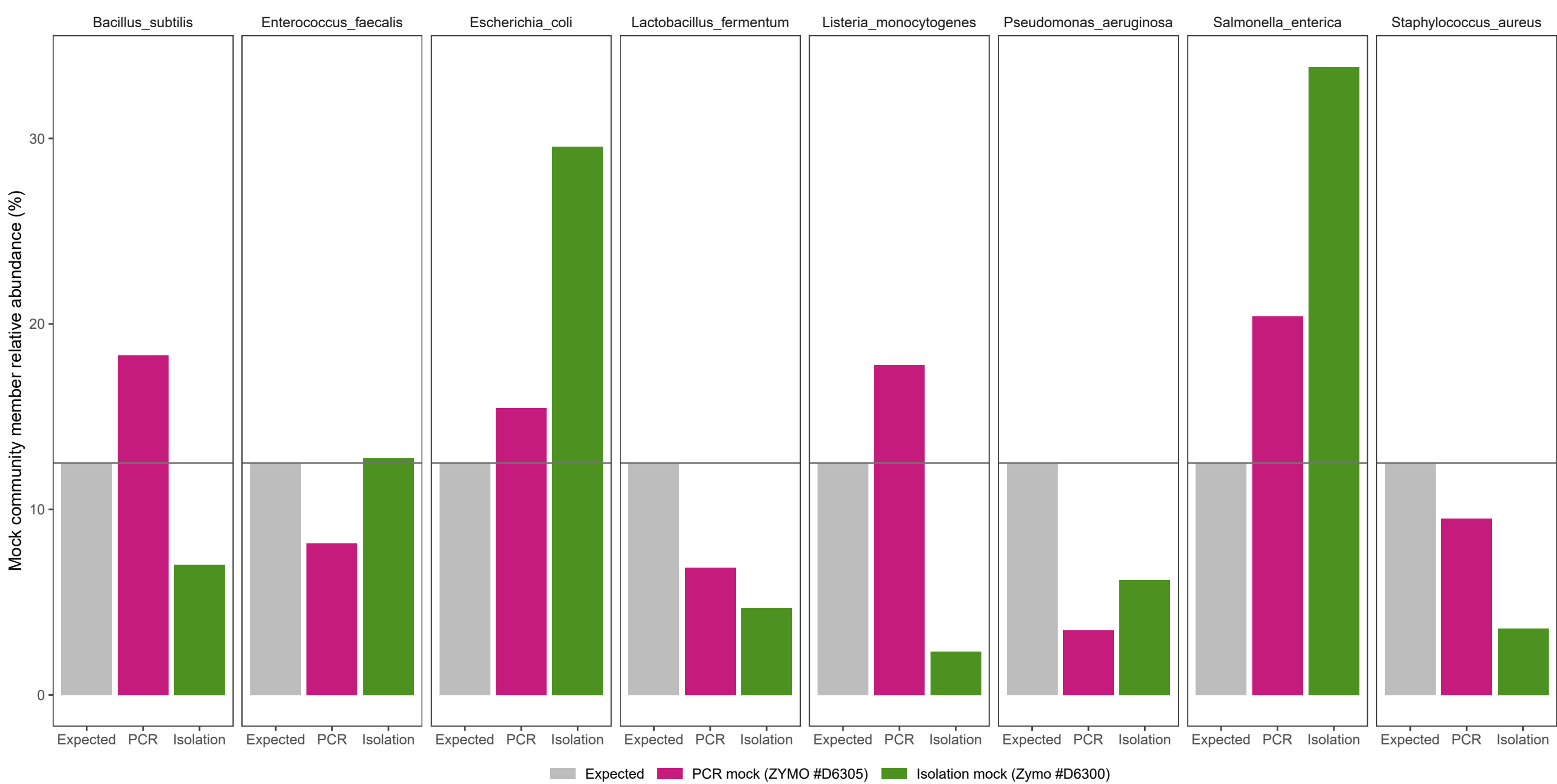

Supplement: Supplementary file 7 — Additional file 7: Figure S1. Boxplot showing the relative abundance of ASVs with 100% identity to the 16S rRNA gene V3-V4 region of mock community members. We used both a DNA isolation mock community (ZymoBIOMICS Microbial Community Standard ZYMO #D6300) and PCR mock community ZymoBIOMICS Microbial Community DNA Standard ZYMO #D6305). [file 12866_2022_2625_MOESM7_ESM.pdf]

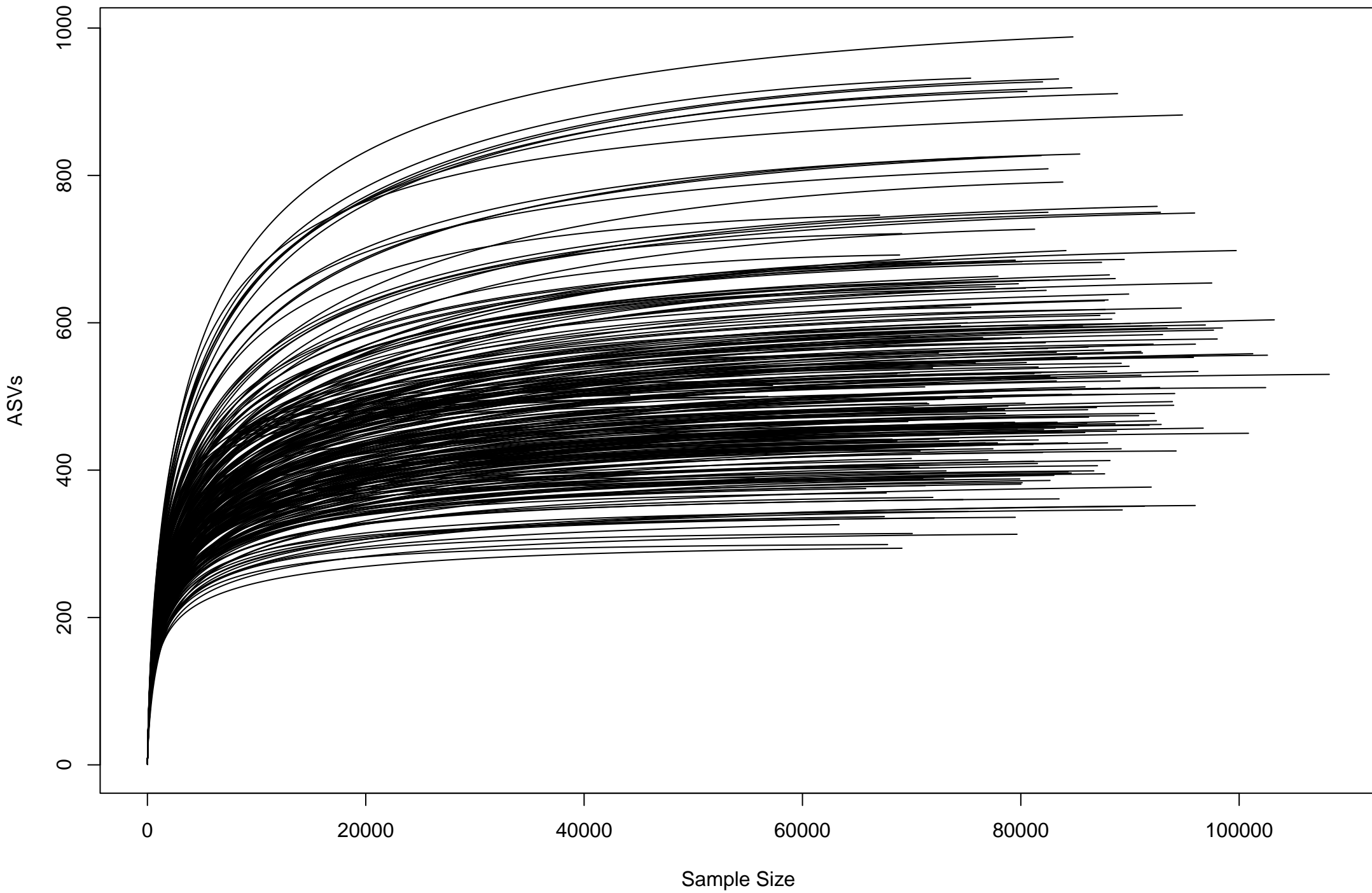

Supplement: Supplementary file 8 — Additional file 8: Figure S2. Rarefaction curves for all samples. Constructed with Vegan function rarecurve. [file 12866_2022_2625_MOESM8_ESM.pdf]

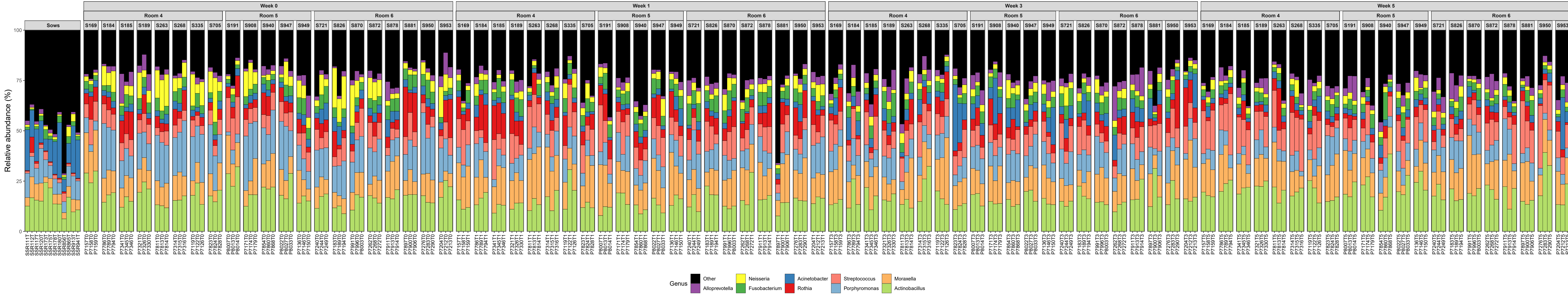

Supplement: Supplementary file 9 — Additional file 9: Figure S3. Stacked barplot showing the abundance of the most abundant genera in all samples. [file 12866_2022_2625_MOESM9_ESM.pdf]

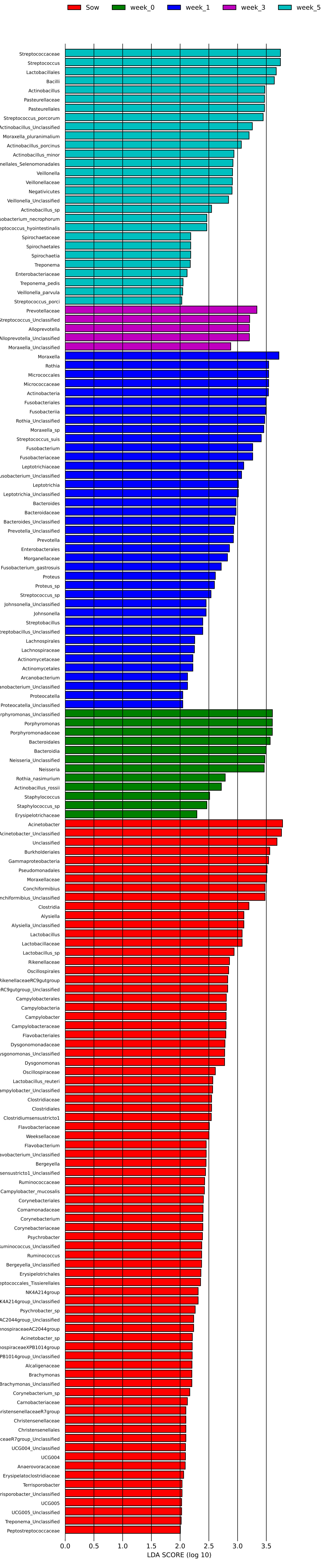

Supplement: Supplementary file 10 — Additional file 10: Figure S4. LEfSe analysis on taxa association with timepoints. [file 12866_2022_2625_MOESM10_ESM.pdf]

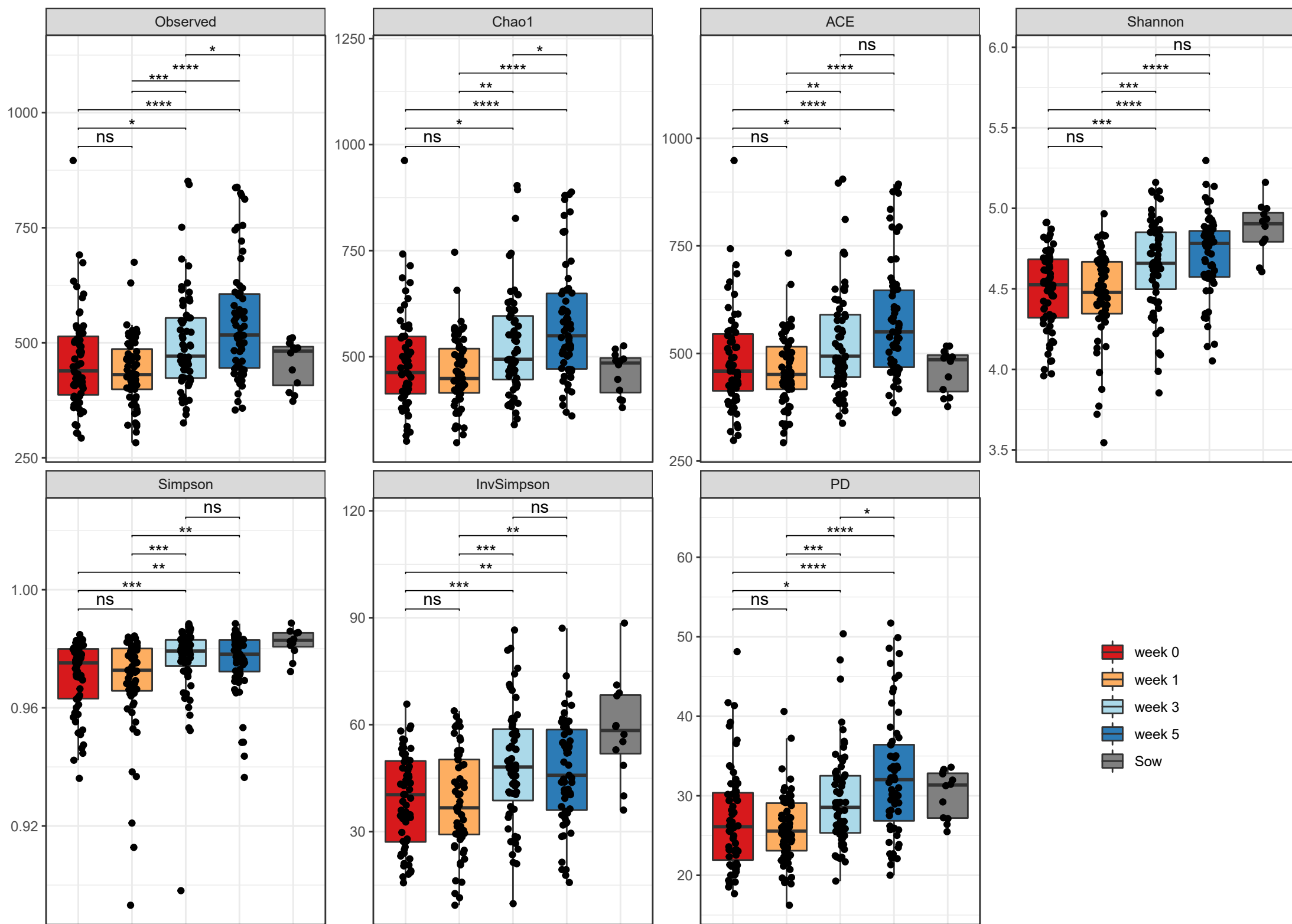

Supplement: Supplementary file 12 — Additional file 12: Figure S6. Boxplots of different alpha diversity measures per timepoint. [file 12866_2022_2625_MOESM12_ESM.pdf]
