# Supplementary material for: Environmental and maternal factors shaping tonsillar microbiota development in piglets
Source: BMC Microbiol. 2022 Sep 26;22:224. doi: 10.1186/s12866-022-02625-8 (PMC9513891; doi:10.1186/s12866-022-02625-8)
Supplement: Supplementary file 11 — Additional file 11: Figure S5. Comparison of S. suis and S. porcorum abundance at different timepoints in the present study and Pena Cortes et. al. 2018 (NCBI BioProject PRJNA391812). S. suis abundance decreases before and across weaning, while S. porcorum increases in abundance at weaning. The higher number of zero-counts found in the dataset of Cortes et al. is in part due to larger variation in sequencing depth. 12 samples had less than 1000 reads. [file 12866_2022_2625_MOESM11_ESM.pdf]

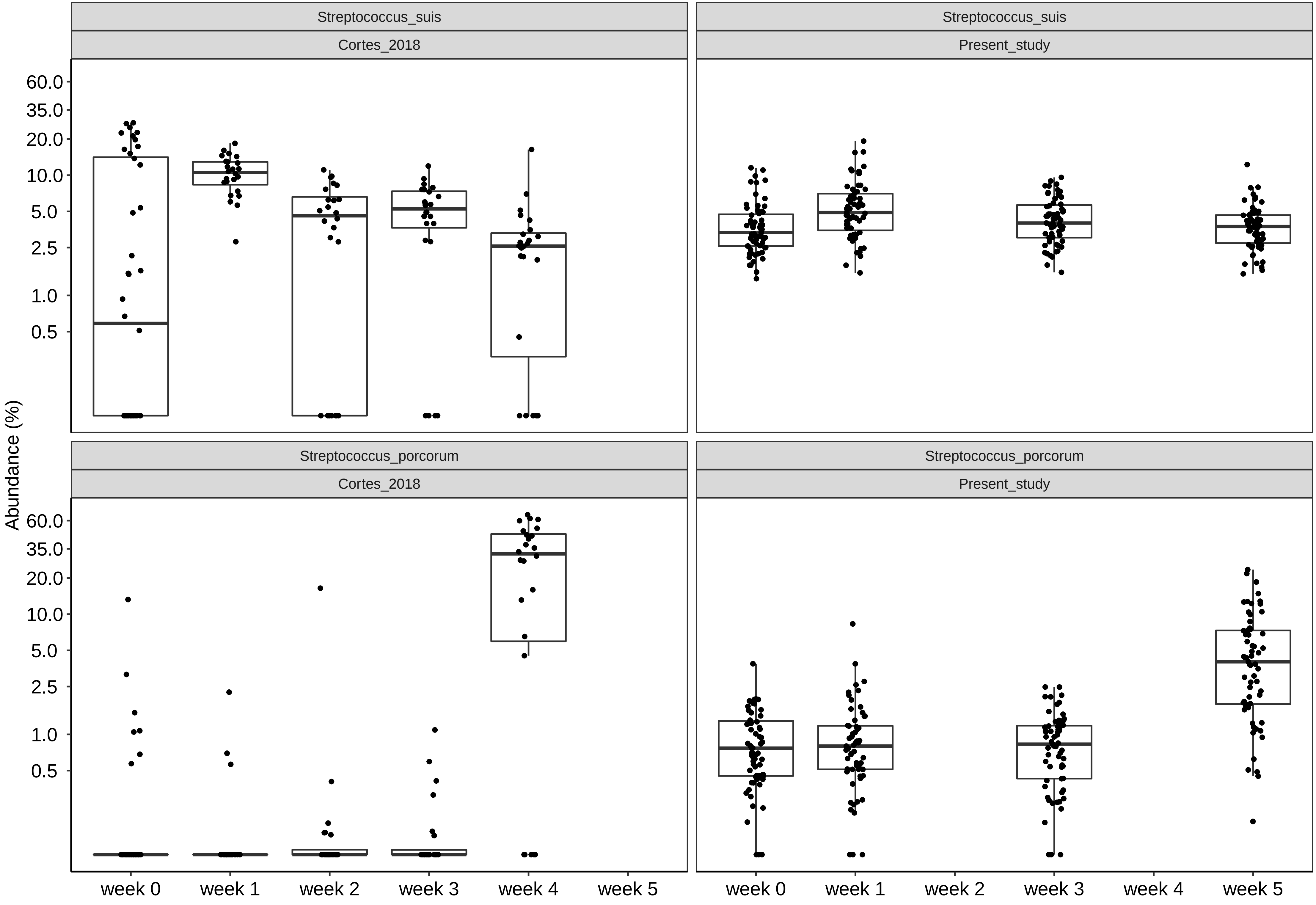
Streptococcus_suis
Cortes_2018
Streptococcus_suis
Present_study
Streptococcus_porcorum
Cortes_2018
Streptococcus_porcorum
Present_study
Abundance (%)
60.0
35.0
20.0
10.0
5.0
2.5
1.0
0.5
week 0
week 1
week 2
week 3
week 4
week 5
